# The effects of an integrated supportive care intervention on quality of life outcomes in outpatients with breast and gynecologic cancer undergoing chemotherapy: Results from a randomized controlled trial

**DOI:** 10.1002/cam4.2196

**Published:** 2019-05-21

**Authors:** Nadja Klafke, Cornelia Mahler, Cornelia von Hagens, Lorenz Uhlmann, Martina Bentner, Andreas Schneeweiss, Andreas Mueller, Joachim Szecsenyi, Stefanie Joos

**Affiliations:** ^1^ Department of General Practice and Health Services Research University Hospital Heidelberg Heidelberg Germany; ^2^ Department of Nursing Institute for Health Sciences University Hospital Tuebingen Tuebingen Germany; ^3^ Division of Naturopathy and Integrative Medicine Department of Gynaecological Endocrinology and Reproductive Medicine University Womens’ Hospital Heidelberg Heidelberg Germany; ^4^ Institute of Medical Biometry and Informatics University of Heidelberg Heidelberg Germany; ^5^ Division Gynaecologic Oncology National Center for Tumor Diseases University Hospital Heidelberg Heidelberg Germany; ^6^ Womens’ Clinic Community Hospital Karlsruhe Karlsruhe Germany; ^7^ Institute of General Practice and Interprofessional Care University Hospital Tuebingen Tuebingen Germany

**Keywords:** breast cancer, chemotherapy, complementary therapies, gynecologic cancer, health services research, nursing intervention, patient‐reported outcomes, quality of life, self‐care, supportive care

## Abstract

The aim of the *Co*mplementary *N*ursing in *G*ynecologic *O*ncology study was to investigate the effects of a complex, nurse‐led, supportive care intervention using Complementary and Integrative Medicine (CIM) on patients’ quality of life (QoL) and associated patient‐reported outcomes. In this prospective, pragmatic, bicentric, randomized controlled trial, women with breast or gynecologic cancer undergoing a new regimen of chemotherapy (CHT) were randomly assigned to routine supportive care plus intervention (intervention group, IG) or routine care alone (control group, CG). The intervention consisted of CIM applications and counseling for symptom management, as well as CIM information material. The primary endpoint was global QoL measured with the EORTC‐QLQ‐C30 before and after CHT. Mixed linear models considering fixed and random factors were used to analyze the data. In total, 126 patients were randomly assigned into the IG and 125 patients into the CG (median age 51 years). The patients’ medical and socio‐demographic characteristics were homogenous at baseline and at follow‐up. No group effects on QoL were found upon completion of CHT (estimate −1.04 [−4.89; 2.81]; *P *= 0.596), but there was a significant group difference in favor of the IG 6 months later (estimate 6.643 [1.65; 11.64]; *P *= 0.010). IG patients did also experience significant better emotional functioning (*P *= 0.007) and less fatigue (*P *= 0.027). The tested supportive intervention did not improve patients’ QoL outcomes directly after CHT (T3), but was associated with significant QoL improvements when considering the change from baseline to the time point T4, which could be assessed 6 months after patients’ completion of CHT. This delayed effect may have resulted due to a strengthening of patients’ self‐management competencies.

## 1 INTRODUCTION

The management of breast and gynecologic cancer patients’ care and treatment is complex and should recognize patient‐reported outcomes (PROs), which are endpoints that complement clinical outcomes, and can be directly experienced and self‐reported by the patients themselves. Patients report high levels of unmet needs and prefer to uptake self‐care methods from the spectrum of Complementary and Integrative Medicines (CIMs)^1^ to alleviate and self‐manage their symptoms often resulting of burdensome treatment phases like chemotherapy (CHT).^2^ The majority of cancer outpatients,^3^ and up to 80% of breast cancer survivors,^4^ complement their conventional cancer treatment with CIM. These numbers are startling, as a majority of patients do not talk about it in clinical consultations^5^ even though there is risk potential of some CIM therapies (eg, phytotherapeutics, supplements) due to interaction with standard therapies.^6^ Cancer patients often rely on healthcare advice from family and friends, or services from other non‐medical healthcare providers, which may be associated with further risks and high costs.^7^ Consequently, there is a need for safe, effective, and feasible cancer care deliveries including evidence‐based CIM applications and counseling to guarantee patients’ safety and address patients’ unmet complementary needs.

Integrating CIM methods in oncological care has resulted in positive effects on PROs^8^, ^9^ and is supported by international clinical guidelines.^10^, ^11^ Meta‐analyses indicate that, for instance, acupuncture and acupressure reduce nausea and pain,^12^ and aromatherapy has the potential to alleviate sleep and anxiety disorders.^13^ There is also recognized evidence that mind‐body‐methods like yoga and meditation increase patients’ quality of life (QoL), and reduce fatigue and distress.^14^ Patients often choose to communicate initially with their oncology nurses about their symptomatic burden and interest for CIM applications,^15^, ^16^ which is understandable as oncology nurses have closest and trustful relationships with the patients. Research indicates that oncology nurses have a positive and open attitude towards the integration of CIM methods into cancer care, but they are often struggling with structural and educational deficits.^16^, ^17^ Accordingly, there is a need to implement healthcare structures focusing on how nurses can respond to cancer patients’ complementary needs.

Research has highlighted that nursing interventions are effective in supporting patients during CHT,^15^ however, the effect of a nurse‐led supportive care packet including CIM tailored to patients’ needs during CHT, has not been shown so far. As oncology nurses represent one of the largest group of health professionals worldwide,^18^, ^19^ and the healthcare provision for oncology patients will continue to rise, integrated concepts of oncological care and treatment, especially for outpatients, will become more important.

In order to assess whether outpatients undergoing CHT benefit from a nurse‐led, supportive care intervention using CIM, the prospective, pragmatic, bicentric, randomized controlled study labeled CONGO (*Co*mplementary *N*ursing in *G*ynecologic *O*ncology) was conducted.^20^

## 2 METHODS

### 2.1 Patients

Details about the study design were published with the study protocol,^20^ designed and reported to meet CONSORT requirements.^21^ The study protocol was approved by the ethics committees of the University of Heidelberg (S‐008/2014) and the State Medical Council of Baden‐Wuerttemberg (B‐F‐2014‐037), Germany. Written informed consent was obtained for intervention participants and control participants prior to study entry.

From 31 July 2014 until 9 February 2016, all breast and gynecologic cancer patients who were scheduled for a new regimen of CHT were eligible to participate in this randomized controlled trial conducted at the National Center for Tumor Diseases (NCT) Heidelberg, and at the Community Hospital Karlsruhe (SKK), Germany. There was no age restriction for adults, and patients were allowed to participate in trials. Exclusion criteria were insufficient knowledge of the German language, cognitive disabilities and inability to give informed consent.

### 2.2 Randomization

Randomization was done with stratified block randomization, using the professional online randomization service *randomizer.at*. Patients were stratified at randomization by CHT treatment intention (curative, palliative) and participating center (NCT, SKK). The patients were randomly allocated (in a 1:1 ratio) to either routine care plus intervention (intervention group [IG]) or routine care (control group [CG]). Blinding was not possible, as the intervention could not be performed blindly.

### 2.3 Procedures

#### 2.3.1 Intervention group

Patients in the IG received CHT with supportive therapy according to the clinics’ guidelines together with the CIM nurse‐led care. This means that based upon the patients’ symptomatic burden and preferences, patients were offered naturopathic applications from the supportive care package in addition to routine care. For example, if a patient suffered of pain, she was offered an Aconite or Solum oil application, or a Melissa oil abdominal rhythmical massage, or a Liver oil upper abdomen rhythmical massage in the outpatient clinic. Based upon their needs, patients also received standardized guidelines on the CIM interventions, so that the patients were able to follow these instructions at home between the CHT cycles, for managing their own symptoms. Patients were comprehensively and regularly counseled by the nurse, and patients were handed out a brochure and CD with more evidence‐based information on CIM.

Further details of the intervention, qualification and training of the nurses, and type and frequency of counseling were reported elsewhere.^22^ Due to possible longer palliative CHTs, the maximum time of the intervention was set to 24 weeks.

#### 2.3.2 Control group

The CHT and supportive therapy plan according to the clinic's guidelines was not changed for the patients in the CG.^23^

### 2.4 Data collection

#### 2.4.1 Recruitment and data collection

Patients’ medical and socio‐demographic characteristics were documented before the CHT (time point of randomization [T0]). PROs were collected at T1 (start of CHT, start of intervention), T2 (mid of CHT treatment, max. after 12 weeks), and T3 (end of intervention, max. after 24 weeks). Follow‐up assessment T4 was conducted 6 months after T3. Patients self‐reported the primary outcome on paper‐based questionnaires and weekly in the patient diary. Data was collected at the participating cancer centers and then transferred to the data management of the University Hospital Heidelberg.

### 2.5 Outcome measures

The primary endpoint was QoL at the end of CHT (T3). Secondary endpoints were QoL at T4 as well as the functional and symptom scales of the EORTC‐QLQ‐C30 at T3 and T4. All other PROs (eg, self‐efficacy, patient competence) were reported separately.

To assess QoL, the EORTC‐QLQ‐C30 was applied due to good psychometric properties.^24^, ^25^ It has been validated in German^26^ as well as with reference groups^27^ and consists of nine symptom scales, five functional scales, and the global QoL. According to the EORTC Scoring Manual,^28^ the latter is measured by two items on a 7‐point Likert scale ranging from 1 to 7, and then transformed linearly into a score between 0 and 100 points.

The five functional scales (physical functioning, role functioning, emotional functioning, cognitive functioning, social functioning) are measured with 4‐point Likert scales with higher scores representing higher functioning. In contrast, higher scores on the nine symptom scales (fatigue, nausea/vomiting, pain, dyspnea, insomnia, appetite loss, constipation, diarrhea, financial difficulties) measured with 3‐ranged Likert scales represent higher burden.

### 2.6 Statistical analysis

Statistical Analysis Software (version 9.4; SAS Institute, Cary, NC) was used for all analyses. Baseline group differences were assessed using descriptive measures combined with *t*‐tests or chi‐squared tests, as appropriate. The transformed score of the combination of the global QoL of the EORTC‐QLQ‐C30 was applied as the primary outcome and base for sample size calculation. To detect *d *=* *0.4 with a power of 1 − β = 80% using a two‐sample *t*‐test at a two‐sided significance level α = 5%, a total of 200 patients for the randomized arm (100 per group) were required.^20^, ^24^

The intention‐to‐treat (ITT) population was used in the primary model where all patients were included in the group they were randomized to. The data were hierarchically arranged in two levels: observations (level 1) measured at different time points were nested in patients (level 2). Therefore, mixed linear models (MLMs) were applied to compare both groups on the primary and secondary outcomes. The treatment group, time of measurement, the interaction of treatment group and time, the QoL baseline score, and the stratification variables tumor stage and cancer center were included as fixed effects. Additionally, a random intercept (for the repeated measurements per patient) was included.

The secondary endpoints were analyzed using MLMs as well. All analyses were then repeated using the per protocol (PP) set excluding patients with major protocol violations. A *P* < 0.05 was considered significant. As predefined in our study protocol,^20^ only the results of the primary analysis are to be interpreted in a confirmatory manner; and when results of the secondary analyses are proven to be significant, these findings will be interpreted exploratory. Finally, for interpreting clinically relevant differences, the guidelines by Cocks et al.^24^ were applied.

## 3 RESULTS

### 3.1 Patients

**Figure 1:**
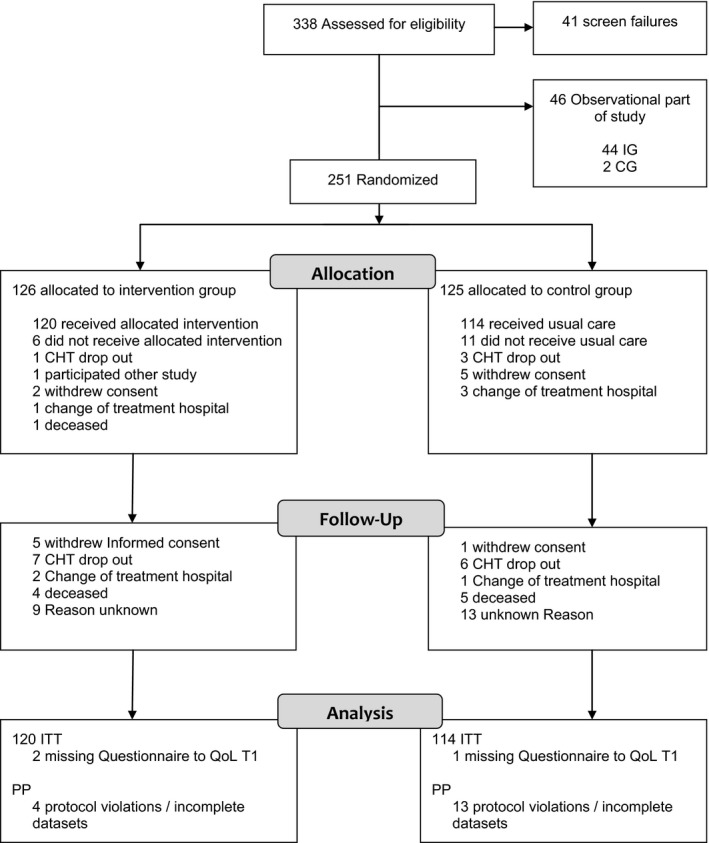
Study flow of the *Co*mplementary *N*ursing in *G*ynecologic *O*ncology‐study. CG, control group; CHT, chemotherapy; IG, intervention group; ITT, intention‐to‐treat; PP, per‐protocol; QoL, quality of life

Descriptive patient data and reasons for patients’ withdrawal is shown in the CONSORT diagram (see Figure 1). The response rate was 87% and 251 patients consented to be randomly allocated to intervention (IG) or CG. The medical and socio‐demographic characteristics of all randomized patients are reported in Table 1.

**Table 1:** Patient characteristics (medical and socio‐demographic)

	IG (n = 120)	CG (n = 114)	Total (N = 234)	*P*
Age				
N	120	114	234	0.631
Mean ± SD	52.6 ± 12.3	51.8 ± 11.4	52.2 ± 11.9	
Median	51.0	51.5	51.0	
Cancer diagnosis				
Breast	101 (84.2%)	96 (84.2%)	197 (84.2%)	0.993
Gynecologic (ovaries, uterus, cervix or other)	19 (15.8%)	18 (15.8%)	37 (15.8%)	
Recurrent				
Yes	11 (9.2%)	11 (9.6%)	22 (9.4%)	0.899
No	109 (90.8%)	103 (90.4%)	212 (90.6%)	
Cancer center				
University hospital	67 (55.8%)	67 (58.8%)	134 (57.3%)	0.650
Community hospital	53 (44.2%)	47 (41.2%)	100 (42.7%)	
Intention of chemotherapy				
Curative	102 (85.0%)	99 (86.8%)	201 (85.9%)	0.686
Palliative	18 (15.0%)	15 (13.2%)	33 (14.1%)	
Postoperative chemotherapy^a^				
Yes	45 (37.5%)	39 (34.2%)	84 (35.9%)	0.600
No	75 (62.5%)	75 (65.8%)	150 (64.1%)	
Preoperative chemotherapy^a^				
Yes	58 (48.3%)	60 (52.6%)	118 (50.4%)	0.511
No	62 (51.7%)	54 (47.4%)	116 (49.6%)	
Radiochemotherapy				
Yes	3 (2.5%)	2 (1.8%)	5 (2.1%)	0.157
No	117 (97.5%)	112 (98.2%)	229 (97.9%)	
Immunotherapy^b^				
Yes	21 (17.5%)	33 (28.9%)	54 (23.1%)	0.038^d^
No	99 (82.5%)	81 (71.1%)	180 (76.9%)	
Hormonal therapy^c^				
Yes	1 (0.8%)	4 (3.5%)	5 (23.1%)	0.157
No	119 (99.2%)	110 (96.5%)	229 (76.9%)	
Other treatments				
Yes	2 (1.7%)	2 (1.8)	4 (1.7%)	0.959
No	118 (98.3%)	112 (98.2%)	230 (98.3%)	
Place of residence				
Metropolitan city (>100 000 inhabitants)	34 (28.3%)	29 (27.6%)	63 (28.0%)	0.977
Small town (20 000‐100 000 inhabitants)	26 (21.7%)	22 (21.0%)	48 (21.3%)	
Countryside (<20 000 inhabitants)	60 (50.0%)	54 (51.4%)	114 (50.7%)	
Missing	0	9	9	
Marital status				
Single	12 (10.3%)	13 (11.7%)	25 (11.0%)	0.033^d^
Married/living with a partner	89 (76.0%)	77 (69.4%)	166 (72.8%)	
Divorced/separated	5 (4.3%)	16 (14.4%)	21 (9.2%)	
Widowed	11 (9.4%)	5 (4.5%)	16 (7.0%)	
Missing	3	3	6	
Place of birth				
Germany	109 (91.6%)	100 (87.7%)	209 (89.7%)	0.330
In another country	10 (8.4%)	14 (12.3%)	24 (10.3%)	
Missing	1	0	1	
Attitude towards CIM				
N	104	100	204	0.636
Mean ± SD	8.1 ± 1.9	8.2 ± 1.8	8.1 ± 1.8	
Experience with CIM				
Yes	59 (50.4%)	38 (34.2%)	97 (42.5%)	0.013^d^
No	58 (49.6%)	73 (65.8%)	131 (57.5%)	
Missing	3	3	6	

Abbreviations: CG, control group; CIM, Complementary and Integrative Medicine; IG, intervention group.

a Administered chemotherapy treatments were: Carboplatin, Paclitaxel, Docetaxel, Epirubicin, Cyclophosphamid, Gemcitabin, Eribulin, Nab‐Paclitaxel, Doxorubicin, Topotecan, Cisplatin, Methotrexat, 5‐Fluorouracil, and other study regimes.

b Administered immunotherapies were: Trastuzumab, Bevacizumab, Pertuzumab.

c Administered hormonal therapies were: Tamoxifen.

d Group difference, *P *<* *0.05.

### 3.2 Primary outcome: global QoL measured with the EORTC‐QLQ‐C30

**Figure 2:**
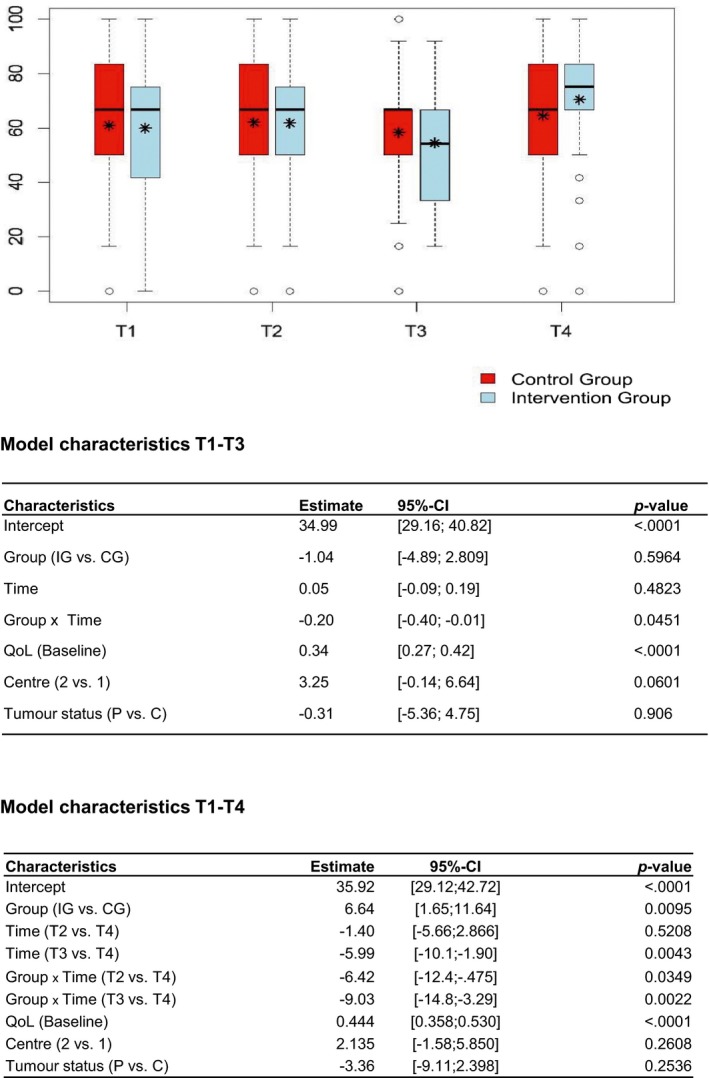
Process of the HRQoL (global domain of the EORTC‐QLQ‐C30), T1‐T4. The * represents the mean (value of the HRQoL).The _ represents the median

Overall development of patients’ global QoL is shown in Table 2 and Figure 2 for the ITT population (N = 231). At baseline, the global QoL levels did not differ between groups (*P *= 0.734). The QoL levels remained constant till T2, and decreased at T3 in both groups. Accordingly, no significant group effect was found for QoL at T3 defined as the main time point (effect estimate −1.04 [−4.89; 2.81]). At T4 (6 month follow‐up), the QoL levels had increased in both groups, but were more pronounced in the IG compared with the CG (*P *=* *0.051). An estimated significant group effect of 6.643 [1.65; 11.64] (*P *=* *0.010), indicating a difference between the two groups IG and CG in favor of the IG, was found in the secondary analysis for the global QoL levels considering the change from baseline (T1) to follow‐up (T4).

**Table 2:** Course of the primary outcome Global Health Status (EORTC‐QLQ‐C30)

Outcome (EORTC‐QLQ‐C30)	RA‐study arm	N = 231	Mean (SD) T1	N = 214	Mean (SD) T2	N = 199	Mean (SD) T3	N = 183	Mean (SD) T4
Global health status	IG	118	59.9 ± 22.9	109	61.8 ± 19.0	98	54.5 ± 20.3	96	70.4 ± 19.8
	CG	113	60.9 ± 23.0	105	62.0 ± 21.5	101	58.3 ± 19.7	87	64.5 ± 21.1^a^

Abbreviations: CG, control group; IG, intervention group; RA, randomized study arm.

a Group difference, *P *<* *0.05.

Sensitivity analyses were performed testing the robustness of the observed effects. Overall 214 patients could be included in the PP analysis, the other patients were excluded due to protocol violations (see Figure 1). The estimated group effects on the primary outcome in the PP analyses were similar compared to the effects observed in the ITT analyses: −2.11 [−6.10; 1.891] (*P *=* *0.30) at T3, and 6.394 [1.142; 11.65] (*P *<* *0.018) at T4. The sensitivity analyses where missing data were imputed led to very similar results, too.

### 3.3 Functional and symptom scales of the EORTC‐QLQ‐C30

In the univariate analyses, group differences were observed for other domains of the EORTC‐QLQ‐C30 reported in Table 3. At T4, significant group differences in favor of the IG were found for fatigue (*P *=* *0.030), emotional functioning (*P *=* *0.011), and dyspnea (*P *=* *0.048). The first two findings were further confirmed by the multivariate analysis using the MLMs approach including the same fixed and random effects as in the primary analysis: patients from the IG experienced significant lower fatigue levels (effect estimate: −7.04 [−13.2; −.840], *P *=* *0.027), and better emotional functioning (effect estimate: 8.196 [2.328; 14.07], *P *=* *0.007). Only small group effects were measured with the EORTC‐QLQ‐C30 at the measurement time points T3 or T4. There was no group effect for dyspnea, presumably because the baseline values were not equal between the groups (*P *=* *0.042).

**Table 3:** Descriptive process of the secondary outcomes of the EORTC‐QLQ‐C30 domains

Outcome (EORTC‐QLQ‐C30 domains)	RA‐study arm	N	Mean (SD) T1	N	Mean (SD) T2	N	Mean (SD) T3	N	Mean (SD) T4
Physical functioning	IG	120	85.1 ± 19.3	107	75.3 ± 20.2	120	68.7 ± 23.5	96	80.9 ± 21.6
	CG	113	82.6 ± 20.2	106	70.7 ± 21.7	114	67.3 ± 22.6	88	77.0 ± 21.4
Role functioning	IG	120	64.3 ± 32.5	109	53.1 ± 30.4	98	44.4 ± 29.3	96	68.2 ± 26.5
	CG	111	61.7 ± 34.1	106	54.7 ± 27.3	101	45.5 ± 30.0	88	61.7 ± 30.7
Emotional functioning	IG	120	50.0 ± 26.0	108	62.8 ± 22.3	99	59.1 ± 25.4	96	65.9 ± 25.6^a^
	CG	114	50.7 ± 26.5	106	64.2 ± 22.4	101	56.5 ± 23.0	88	56.3 ± 24.7^a^
Cognitive functioning	IG	120	77.4 ± 24.8	109	72.2 ± 24.2	99	70.7 ± 26.6	96	73.4 ± 24.6
	CG	114	77.2 ± 25.0	106	72.2 ± 23.5	101	70.3 ± 25.5	88	68.8 ± 26.7
Social functioning	IG	120	59.3 ± 31.3	109	62.1 ± 29.4	99	54.5 ± 29.6	96	69.1 ± 27.9
	CG	113	58.6 ± 29.6	106	59.7 ± 27.6	101	55.1 ± 29.5	88	63.6 ± 26.6
Fatigue	IG	120	37.0 ± 29.1	109	50.2 ± 25.8	98	56.3 ± 26.9	96	36.2 ± 25.7^a^
	CG	113	41.2 ± 28.2	106	51.5 ± 24.6	101	59.4 ± 25.7	88	45.1 ± 29.1^a^
Nausea/vomiting	IG	120	8.9 ± 18.6	108	13.1 ± 20.1	98	13.1 ± 20.2	96	5.4 ± 14.6
	CG	113	8.0 ± 17.6	106	13.5 ± 19.3	101		88	4.9 ± 13.6
Pain	IG	120	34.0 ± 32.7	109	26.8 ± 28.7	98	32.8 ± 33.7	96	27.8 ± 28.4
	CG	113	35.5 ± 30.8	106	29.6 ± 28.0	101	35.1 ± 30.8	88	30.5 ± 29.8
Dyspnea	IG	120	15.3 ± 25.2^a^	109	33.3 ± 31.8^a^	97	45.0 ± 32.3^a^	95	23.2 ± 28.0^a^
	CG	112	22.3 ± 27.4^a^	104	42.9 ± 30.0^a^	100	49.3 ± 33.3^a^	87	32.2 ± 33.1^a^
Insomnia	IG	119	44.8 ± 35.1	107	46.1 ± 34.5	98	50.0 ± 33.6	96	40.3 ± 31.7
	CG	113	44.0 ± 33.7	106	41.8 ± 33.8	101	48.8 ± 35.5	88	48.1 ± 35.7
Appetite loss	IG	119	21.8 ± 30.5	108	20.1 ± 26.5	97	20.3 ± 26.6	96	10.1 ± 21.1
	CG	113	21.2 ± 30.9	105	20.0 ± 28.3	101	20.5 ± 30.9	88	13.6 ± 25.1
Constipation	IG	119	15.1 ± 28.7	108	26.9 ± 32.0	98	24.5 ± 30.9	96	11.5 ± 24.1
	CG	113	12.1 ± 25.2	104	22.1 ± 31.4	100	19.0 ± 29.7	88	9.8 ± 22.1
Diarrhea	IG	119	12.0 ± 22.4	108	18.5 ± 31.4	97	20.3 ± 29.9	96	10.8 ± 21.9
	CG	112	8.9 ± 20.0	105	24.8 ± 34.3	101	27.4 ± 37.8	85	7.8 ± 21.6
Financial problems	IG	118	21.8 ± 31.5	109	21.1 ± 10328.6	99	24.9 ± 30.2	95	25.6 ± 29.4
	CG	114	23.7 ± 31.3	103	22.7 ± 25.2	101	25.7 ± 29.4	88	27.3 ± 30.1

Abbreviations: CG, control group; IG, intervention group; RA, randomized study arm.

a Group difference, *P *<* *0.05.

## 4 DISCUSSION

Our results demonstrate that the administration of supportive care including nurse‐led CIM therapies, improves QoL compared to routine care in breast and gynecologic cancer patients 6 months after completion of CHT, but not directly after CHT as initially hypothesized.

The findings indicate that patients in both groups experienced an increase in their QoL in the timespan from completion of CHT (T3) until 6 months later (T4). During this follow‐up phase, an increase of QoL levels was measured in both groups, but more in the IG. Within the latter group, the improvement of 15.9 scores corresponded to a medium effect,^24^, ^29^ further demonstrating the health benefits of the tested intervention. Even though the significant QoL group difference between IG and CG measured at T4 is smaller than 10 points, and therefore does not reach the threshold to a medium effect,^24^, ^30^ the effect can be interpreted as clinically relevant though, as the supportive intervention was safe and addressed patients’ needs. Surprisingly, likewise as with the global QoL, the levels of fatigue and emotional functioning were stable in the IG and CG from the start of CHT until the midst of CHT, then a deterioration was assessed in both groups at the end of CHT (T3). At follow‐up (T4), 6 months after completion of CHT, both outcomes improved, but significantly more in the IG as shown by the univariate and multivariate analyses. Likewise as with the global QoL scores, the other two PROs improved, but compared with reference groups such as other chronic patients or people from the general population, the symptom burden was higher and functioning was more impaired.^26^, ^27^

Assessments of patients’ QoL experience have become widely accepted in clinical trials,^31^ as patients’ experience in QoL has been shown to be an important and prognostic factor for overall survival.^32^, ^33^ Consequently, healthcare interventions focusing on QoL that are feasible and can be translated back into clinical healthcare deliveries, also for outpatient care,^29^ are highly needed. To our knowledge, this study is the first randomized trial comparing the effect of a nurse‐led CIM intervention on QoL with routine care in outpatient cancer centers during CHT. To date, supportive interventions including CIM have been tested only within a few studies. These interventions, however, were not specifically integrated to supportive care during CHT,^34^, ^35^ considered exercise as the only intervention element,^35^ primarily considered other PROs like fatigue^8^, ^35^, ^36^, ^37^ or did not consider a nurse‐led approach.^8^, ^9^ The latter aspect, however, should be prioritized as nurses are often involved in patients’ discussions about CIM, but need more education and evidence‐based healthcare structures for conducting patient‐oriented care.^17^

Several aspects need to be considered as to why the integrated nurse‐led CIM approach reported here did not demonstrate QoL benefits in patients completing CHT. CHT is the most burdensome treatment phase for cancer patients,^2^ and oncological rehabilitation studies report that patients’ QoL needs almost a month to recover after primary treatment.^38^ This might be a possible explanation for not having found an effect shortly after the end of CHT. So, it can be hypothesized that the positive effect needs time to develop. This is supported by the finding that the delayed effect was also found for other scales of the EORTC‐QLQ‐C30 as shown for emotional functioning and fatigue. One other reason for the observed delayed effect of the CONGO‐study might lie in the fact that the intervention was administered during patients’ “teachable moments” and could therefore be effective over time. Previous research has indicated that psycho‐educative interventions are most successful for cancer patients and survivors if these are administered during or at the end of a strenuous treatment phase, so that their needs to strengthen their competencies, self‐efficacy, and health behaviors can be early addressed,^39^, ^40^ which is in line with the current findings. All CIM interventions had been standardized for the patient diary,^22^ so that patients could follow the CIM instructions at home and take care of their symptoms themselves.^41^ Preliminary results from the accompanying process evaluation^42^ confirm that patients were highly interested to continue applying the CIM interventions after positive experiences and wished to sustain them. Patients underlined how important it was for them to act autonomously and get back to normal as early as possible. The strong desire of patients to be independent and regain control is supported by many studies,^43^ and in our study we provided patients of the IG with self‐care strategies for the time during and after CHT.

The major strengths of our study are the large sample size and randomization, resulting in comparable groups of female cancer patients. Participating patients demonstrated high compliance and interest throughout the whole study, as well as high adherence with the study protocol. The findings of the study have high external validity as we used routine care as the CG. Thus, it was possible to focus on effectiveness (rather than efficacy) to best represent daily outpatient cancer care in the participating centers. Study quality was demonstrated by high data quality and an overall drop‐out rate of 21%, which can be regarded as normal and not affecting the robustness of the detected effects, as there was still a large sample size at T4 (n* *=* *183).

The generalizability of our study may be limited to the studied population, focusing on adult German female outpatients treated for breast or gynecologic cancer in a University and community hospital. Other limitations may lie in the selection of indications used for the design and implementation of the CIM interventions as part of the study. Future implementation, supportive care and survivorship research might involve other patient groups and refine the symptom clusters, so that patients’ need can be addressed and met even more comprehensively. Further research is needed to investigate the optimal dose of the CIM counseling sessions, the interlinking of training modules for healthcare staff, and the best context conditions for the integration of such CIM supportive therapy interventions.

In conclusion, symptom management is highly relevant for cancer patients undergoing CHT, and the application of evidence‐based CIMs combined with regular counseling provide a safe and benign option, when administered by trained oncology nurses. Oncological centers that prioritize patient‐oriented care may consider integrating evidence‐based CIM healthcare services led by nurses. Our study does demonstrate a positive effect on PROs 6 months after patients completed their CHT, indicating further application and implementation of the tested nurse‐led CIM intervention.

## CONFLICT OF INTEREST

None declared.

## AUTHOR CONTRIBUTIONS

SJ, CH, CM, AS, AM, NK participated in the design and concept of the study. MB participated in the data collection and enrollment of patients. LU was involved in data analysis. NK was involved in literature research, data analysis, data interpretation, and writing of the report. SJ, CM, CH, MB, JS, NK participated in the interpretation of results. All authors have participated in drafting and finalizing the report.
